# Interception of Signaling Circuits of Esophageal Adenocarcinoma Cells by Resveratrol Reveals Molecular and Immunomodulatory Signatures

**DOI:** 10.3390/cancers13225811

**Published:** 2021-11-19

**Authors:** Hardika Dhir, Monica Choudhury, Ketki Patil, Candice Cheung, Adriana Bodlak, Danny Pardo, Asana Adams, Stefano Travaglino, Jose Araque Rojas, S. Balakrishna Pai

**Affiliations:** Wallace H. Coulter Department of Biomedical Engineering, Georgia Institute of Technology and Emory University, 313 Ferst Drive, Atlanta, GA 30332, USA; hardika13@gmail.com (H.D.); mchoudhury165@gmail.com (M.C.); kpatil7@mail.gatech.edu (K.P.); cvcheung@email.sc.edu (C.C.); adrianabodlak@gmail.com (A.B.); dannyp1013@gmail.com (D.P.); asanaadams@hotmail.com (A.A.); stefano.travaglino@gatech.edu (S.T.); jaraque97@gmail.com (J.A.R.)

**Keywords:** esophageal adenocarcinoma, resveratrol, OE33, OE19, FLO-1, apoptosis, caspases, Bcl2, proteomics, flow cytometry

## Abstract

**Simple Summary:**

Adenocarcinoma of the esophagus has been on the rise lately. Increase in mortality due to a paucity of efficacious drugs for this cancer prompted us to discover molecular signatures to combat this malady. To this end, we chose resveratrol—a polyphenol and studied its impact on three esophageal adenocarcinoma cell lines (OE33, OE19 and FLO-1) by multilevel profiling. Here, we show the impact of resveratrol on the viability of the three cell systems studied, at the cellular, molecular level and by proteomic analysis. Impact on programmed cell death pathway resulting in an increase in apoptotic and caspase-positive cells were observed. Decrease in Bcl2. levels and impact on reactive oxygen species (ROS) was also observed. Moreover, proteomic profiling highlighted pivotal differentially regulated signaling molecules. Notably, the downregulation of Ku80 by resveratrol could be harnessed for chemo-radiation therapy to prevent DNA break repair after radiation therapy. Additionally, protein profiling has shed light on molecular and immune-modulatory signatures with implications for discovering novel treatments such as chemo-immunotherapy.

**Abstract:**

Deregulation of signaling pathways due to mutations sets the cell on a path to neoplasia. Therefore, recent reports of increased mutations observed in esophageal tissue reflects the enhanced risk of tumor formation. In fact, adenocarcinoma of the esophagus has been on the rise lately. Increase in mortality due to a paucity of efficacious drugs for this cancer prompted us to discover molecular signatures to combat this malady. To this end, we chose resveratrol—a polyphenol with anticancer property—and studied its impact on three esophageal adenocarcinoma cell lines (OE33, OE19 and FLO-1) by multilevel profiling. Here, we show the impact of resveratrol on the viability of the three adenocarcinoma esophageal cell systems studied, at the cellular level. Furthermore, an analysis at the molecular level revealed that the action was through the programmed cell death pathway, resulting in an increase in apoptotic and caspase-positive cells. The impact on reactive oxygen species (ROS) and a decrease in Bcl2 levels were also observed. Moreover, proteomic profiling highlighted pivotal differentially regulated signaling molecules. The phenotypic effect observed in resveratrol-treated esophageal cells could be due to the stoichiometry per se of the fold changes observed in entities of key signaling pathways. Notably, the downregulation of Ku80 and other pivotal entities by resveratrol could be harnessed for chemo-radiation therapy to prevent DNA break repair after radiation therapy. Additionally, multilevel profiling has shed light on molecular and immune-modulatory signatures with implications for discovering novel treatments, including chemo-immunotherapy, for esophageal adenocarcinomas which are known to be aggressive cancers.

## 1. Introduction

Organs such as the skin and cells of the hematopoietic system are known to be prone to mutations; however, a key report on the assessment of mutations points to the esophagus as an additional organ vulnerable to somatic as well as cancer-related mutations [1]. Ultimately, these accumulated mutations could lead to neoplasia of the esophagus. In fact, more than 400,000 patients with carcinomas of the esophagus are identified every year [2]; moreover, the patient survival rate for this cancer has not improved over years [3]. Studies on esophageal cancers have led to the classification of this neoplasia as squamous carcinomas or adenocarcinomas; additionally, a few mixed types have been identified [4]. Although squamous carcinomas are predominant, lately, there has been an increase in the rate of adenocarcinomas of the esophagus, especially in Western countries [5]. Due to a paucity of efficacious drugs to combat this aggressive type of cancer, there is an urgent need for identification of novel strategies to inhibit this malignancy.

Recently, natural products with anticancer activity have been sought after to treat various types of diseases, including cancers, because of their minimal side effects. Certain plants such as grapevines are known to synthesize non-flavonoid phenolic compounds, these molecules are categorized as phytoalexins and one such compound is resveratrol (3,5,4′-trihydroxy-trans-stilbene). In cervical cancers, this molecule is shown to induce apoptosis as well as downregulate the antiapoptotic molecule Bcl2 [6]. Furthermore, in human neuroblastomas, it causes apoptosis by its action on the mitochondria [7], whereas in pancreatic cancers, its action results in an increase in sensitivity to drugs such as gemcitabine [8]. Additionally, resveratrol has shown chemopreventive activity against various cancers including esophageal cancers; in F344 rats, resveratrol suppressed N-nitrosomethylbenzylamine (NMBA)-induced esophageal tumorigenesis. Furthermore, in U937 cells, resveratrol inhibited progression through S and G2 phases of the cell cycle. Additionally, supplementation of resveratrol led to chemoprevention of metaplasia initiation and carcinogenic progression of esophageal adenocarcinoma [9,10,11].

Resveratrol’s action is multifaceted including modulation of key transcription factors [12]. Furthermore, it is a DNA synthesis inhibitor and enzymatic assays demonstrate its direct action on DNA polymerase [13]. In ovarian cancer cells, resveratrol causes cell death through an ROS-dependent pathway [14]. Additionally, there are reports describing the immunomodulatory effects of resveratrol; it is shown to affect the crosstalk between immune and cancer cells in colon cancer [15] and also exhibits unique action on immune cells as well as the endothelial cells [16]. Although immunomodulation has been shown, further studies on identifying immunomodulatory entities for novel treatment regimens such as chemo-immunotherapy are needed. In the absence of a well-defined pre-clinical model system to study the effect of compounds on adenocarcinomas of the esophagus [17], we sought to study the impact of resveratrol on the viability of the adenocarcinoma of the esophagus using three well characterized cell systems, i.e., OE33, OE19 and FLO-1. By conducting multilevel profiling, such as cellular, molecular and proteomic analysis, we have attempted to discover novel cancer signatures with the aim of developing novel strategies to combat adenocarcinoma of the esophagus—a debilitating aggressive form of cancer.

## 2. Materials and Methods

### 2.1. Cell Line and Materials

Resveratrol was purchased from Sigma-Aldrich. Stock solution of Resveratrol (100 mM in DMSO) was used for the study. Assay kits for Muse flow cytometry were supplied by EMD Millipore (Burlington, MA, USA).

Human esophageal adenocarcinoma cell lines, i.e., OE33, OE19 and FLO-1 cell lines, were purchased from Sigma-Aldrich. OE33 and OE-19 cells were grown in RPMI medium supplemented with 10% fetal bovine serum, 1% Penicillin/Streptomycin and 2 mM glutamine. FLO-1 cells were cultured in DMEM medium supplemented with 10% fetal bovine serum, 1% Penicillin/Streptomycin and 2 mM glutamine. Cultures were incubated at 37 °C in an atmosphere of 5% CO_2_.

### 2.2. Assessment of the Impact of Resveratrol on the Viability of OE33, OE19 and FLO-1 Cells

Impact of resveratrol on cell viability was assessed by employing the published protocols [18,19]. Essentially, in 96-well plates, 5000 cells per well were plated. The assays were done in triplicates. After 24 h, cells were treated with varying concentrations of resveratrol. On incubation at 37 °C for 72 h, the media was aspirated and 200 μL of 10% Cell Counting Kit-8 (CCK8) solution in complete growth medium was added. With an Infinite 200Pro plate reader, the absorbance at 450 nm was measured after incubation at 37 °C for 1.5 h. Viability of cells was expressed as the percentage of cells that were alive in the treatment group compared to the control group. Using the GraphPad, the IC_50_ was determined and the concentrations of resveratrol were plotted versus the values obtained from the CCK8 assay. Three independent experiments were performed for each of the cell lines.

### 2.3. Muse Flow Cytometric Analysis

Flow cytometry experiments were performed as per previously published procedures with some modifications [18,19]. Assays were performed in a 12-well plate in triplicates; cells (62,000/well) were plated. After 24 h of plating, resveratrol was administered at a concentration of 100 μM for OE33, 50 μM for OE19 and 40 μM for FLO-1 (IC_50_ concentration of each cell line) with untreated controls maintained in parallel. After 72 h of incubation at 37 °C in an atmosphere of 5% CO_2_, adherent cells were collected by trypsinization and further analysis was conducted. Cells were pooled together from triplicate treatments and were left on ice until analysis.

The following flow cytometric assays were conducted using a Muse Cell Analyzer: (1) Annexin V assay (MCH100105), (2) Bcl2 Dual detection activation assay (MCH200105), (3) Multicaspase assay (MCH100109) and (4) Oxidative stress assay (MCH100111). Assays were performed as per manufacturer’s protocols (Millipore-Sigma). Essentially, for the Annexin V assay, cells were stained with Annexin V & Dead Cell Reagent for 20 min at room temperature and protected from light. For the Bcl2 activation assay, cells were fixed using the Muse fixation buffer and permeabilized using ice-cold Muse permeabilization buffer. The fixed cells were stained with anti-Bcl2, PECy5 antibody for 30 min at room temperature in the dark. To determine whether caspases are involved, Muse Multicaspase Reagent working solution was added to cells and then incubated for 30 min at 37 °C in an atmosphere of 5% CO_2_. This was followed by Muse Caspase 7-AAD working solution and incubation at room temperature for 5 min, protected from light. Analysis for each assay was done as per the manufacturer’s instructions.

### 2.4. Proteomic Analysis

Proteomic analysis was performed by 2D DIGE and Mass spectrometry, which was performed by Applied Biomics Inc. (Hayward, CA, USA) employing previously published methodology [18,20,21]. OE33, OE19 and FLO-1 cells were treated with resveratrol at their respective IC_50_ concentrations. Control cultures (no treatment) were maintained in parallel. Essentially, for protein profiling, the following procedures were employed.

### 2.5. Protein Lysate Preparation

Cells from control samples and resveratrol-treated samples were collected, washed with 1X PBS and then stored at −80 °C prior to sending the samples to Applied Biomics, (Inc., Hayward, CA, USA) (on dry ice for proteomics analysis). Cells were lysed in 2D cell lysis buffer consisting of 30 mM Tris-HCl-pH 8.8, 7 M urea, 2 M thiourea and 4% CHAPS, and then sonicated on ice and left on a shaker for 30 min at room temperature. Next, the cell suspension was centrifuged at 25,000× *g* for 30 min at 4 °C. The supernatant of the sample was collected and then diluted in 2D-lysis buffer to achieve a protein concentration of 6 mg/mL.

### 2.6. Minimal CyDye Labeling

The CyDye labeling protocol employed was essentially as described previously [18,20,21]. Protein lysate at a concentration of 30 μg was taken and 1.0 μL of diluted CyDye (1:5 diluted with DMF from 1 nmol/μL stock) was added, vortexed and incubated on ice for 30 min in the dark. After the addition of 1.0 μL of 10 mM Lysine to each of the samples, they were incubated for additional 15 min. The samples were labeled with Cy3 and Cy5; IEF and SDS-PAGE was performed as per published protocols [18,20,21]. This was followed by image scan, spot picking and mass spectrometry [18,20]. Essentially, the spots identified represent biological means for each of the cell line. Proteins that were upregulated/downregulated in the treatment group compared to the control were identified. The National Center for Biotechnology Information non-redundant (NCBInr) or Swiss Protein database was used to identify the proteins. A score of C.I.% or Ion C.I.% greater than 95 was considered significant [18,20]. Furthermore, upregulated/downregulated proteins common to all the three cell lines (OE33, OE19 and FLO-1) with comparable fold changes were selected and were subjected to mass spectrometry.

### 2.7. Statistical Analysis

One-way ANOVA analysis with Dunnett’s multiple comparison test analysis, with alpha value set to 0.05 was performed. For the Muse assays, an ANOVA was done with Dunnett’s multiple comparison for both Annexin and MultiCaspase assays. ROS and Bcl2 data analysis was performed by ANOVA. For all the assays, the *p*-value was set to 0.05.

## 3. Results

### 3.1. Resveratrol Impacts Cell Viability of OE33, OE19 and FLO-1 Cells

**Figure 1:**
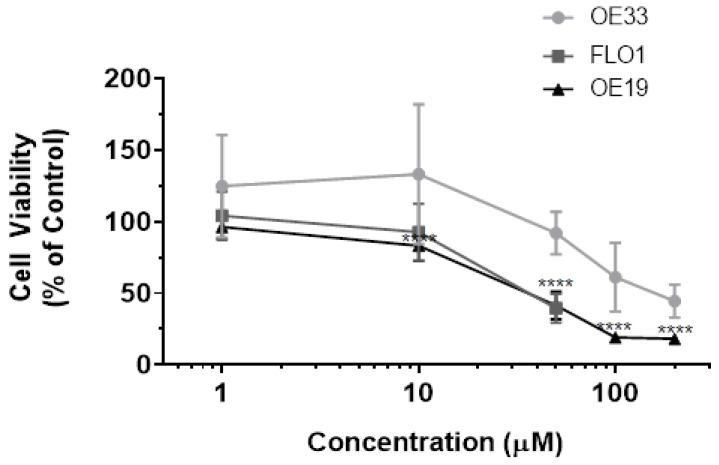
Cytotoxicity of resveratrol on the cells of the adenocarcinoma of the esophagus. OE33 cells (5000/well) were plated in a 96-well plate. After 24 h, cells were treated with various concentrations of resveratrol and incubated for 72 h. The cell viability was assessed by performing the CCK8 assay. The experiments were done in triplicates. The mean of three such independent experiments were calculated. OE19 cells were also treated and processed as described for OE33. Additionally, FLO-1 cells were similarly processed. The percentage of viable cells in the treatment group was calculated by considering cells in each of the control group as 100%. The data points for each cell line are the mean of three such independent experiments. A one-way ANOVA was used using Dunnett’s multiple comparisons test with the Alpha value set to 0.05. **** FLO-1: *p*-value < 0.0001 from 25 μM onwards. OE19: *p*-value < 0.0001 from 10 μM onwards, OE33: significant change seen from 200 μM onwards.

To assess the anticancer potential of resveratrol on the human esophageal adenocarcinomas; OE33, OE19 and FLO-1 cells were treated with various concentrations of resveratrol and incubated for 72 h. Resveratrol exhibited dose-dependent inhibition of cell viability in all the three cell systems, as shown in Figure 1. The inhibition of cell proliferation was assessed by performing the CCK-8 assay. The IC_50_ was calculated to be 100 μM for OE33, 50 μM for OE19 and 40 μM for FLO-1.

### 3.2. Molecular Analysis of Resveratrol’s Action Reveals the Impact on the Programmed Cell Death Pathway in the Three Esophageal Adenocarcinoma Cell Systems (OE33, OE19 and FLO-1) Studied

**Figure 2:**
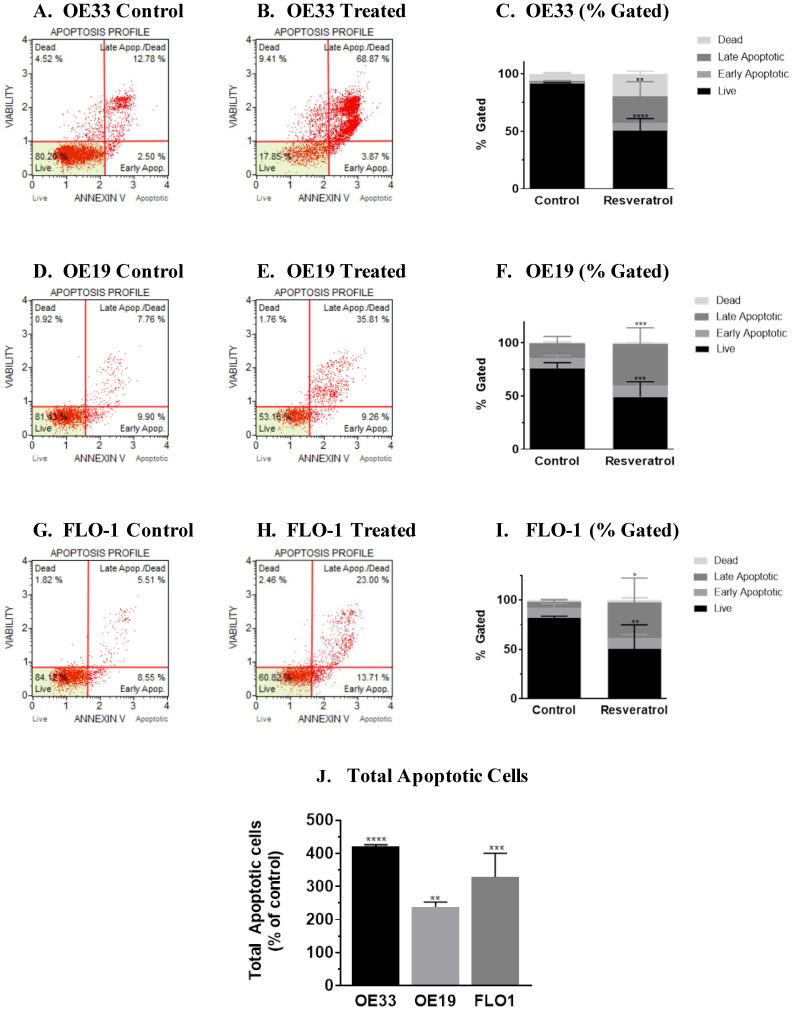
Induction of programmed cell death in resveratrol-treated OE33, OE19 and FLO-1 cells. OE33 cells were seeded at a density of 62,500 cells/well in 12-well plates, treated with resveratrol at an IC_50_ concentration of 100 µM for 72 h. Three independent experiments were performed. (**A**) Representative scatter plot of Muse Annexin assay for control OE33 group. (**B**) Representative scatter plot for Muse Annexin assay for resveratrol-treated OE33 group. (**C**) The profiles (% gated) of the control group and treated group. Similar to OE33 cells, OE19 and FLO-1 cells were treated with resveratrol at the IC_50_ concentration of 50 and 40 μM, respectively. (**D**) Representative scatter plot of four independent trials of the OE19 control group is shown. (**E**) The OE19 resveratrol-treated group. (**F**) The profiles (% gated) of the control group and the treated group of OE19 cells. Four independent trials were also performed for FLO-1 cells. (**G**) Representative scatter plot of the control FLO-1 cells and (**H**) treated FLO-1 group. (**I**) The % gated profiles of the FLO-1 control group and the treated group. (**J**) On normalizing to the control group, the percent of apoptotic cells in the treated group are represented by a bar graph for each cell line. The *p*-value was calculated for each cell line. * Represents *p*-value < 0.05. More asterisks represent values of higher statistical significance.

On observing cell death caused by resveratrol, we proceeded to understand its mechanism of action. Programmed cell death pathway is one of the major pathways induced by anticancer agents. To assess if programmed cell death pathway was induced by resveratrol in each of the cell system, we opted to perform flow cytometric analysis, because this assay is reported to be a sensitive assay. Annexin V Muse flow cytometric assay was performed by treating OE33 cells with the IC_50_ concentration of resveratrol (100 μM) for 72 h and compared with the control group. Representative scatter plots of three independent trials of the control group and the treated group are shown in Figure 2A,B, respectively. The profiles (% gated) of the treated group versus the control group showed an increase in apoptotic population in the treated group, Figure 2C. Similarly, OE19 and FLO-1 cells were treated with resveratrol at an IC_50_ concentration of 50 and 40 μM, respectively, and analyzed by Annexin V Muse flow cytometric analysis. For OE19 cells, representative scatter plots of four independent trials of the control group and the treated group are shown in Figure 2D,E, respectively. The profiles of the treated group versus the control group also showed an increase in apoptotic population in the treated group, Figure 2F. For FLO-1 cells, representative scatter plot of four independent trials of the flow cytometric assay of the control and treated cells are shown in Figure 2G,H. The profiles of the control group versus the treated group showed an increase in apoptotic cells, as seen in Figure 2I. The studies showed that on the administration of resveratrol, there was a significant increase in the total number of apoptotic cells, above 4-fold when compared to the control group in OE33; above 2-fold in OE19 and above 2 to 3-fold in FLO-1 cells when compared to their respective control group, Figure 2J.

### 3.3. Activation of Caspases by Resveratrol in Esophageal Adenocarcinoma Cells

**Figure 3:**
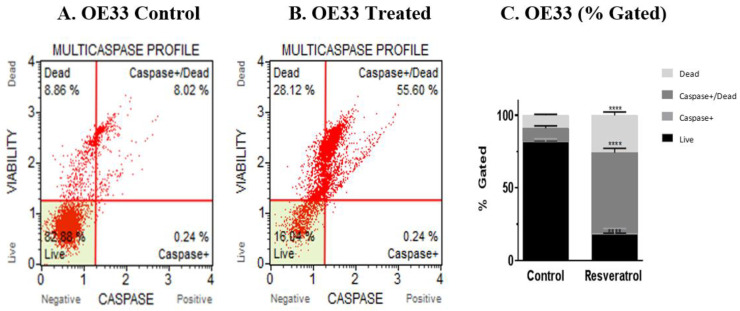

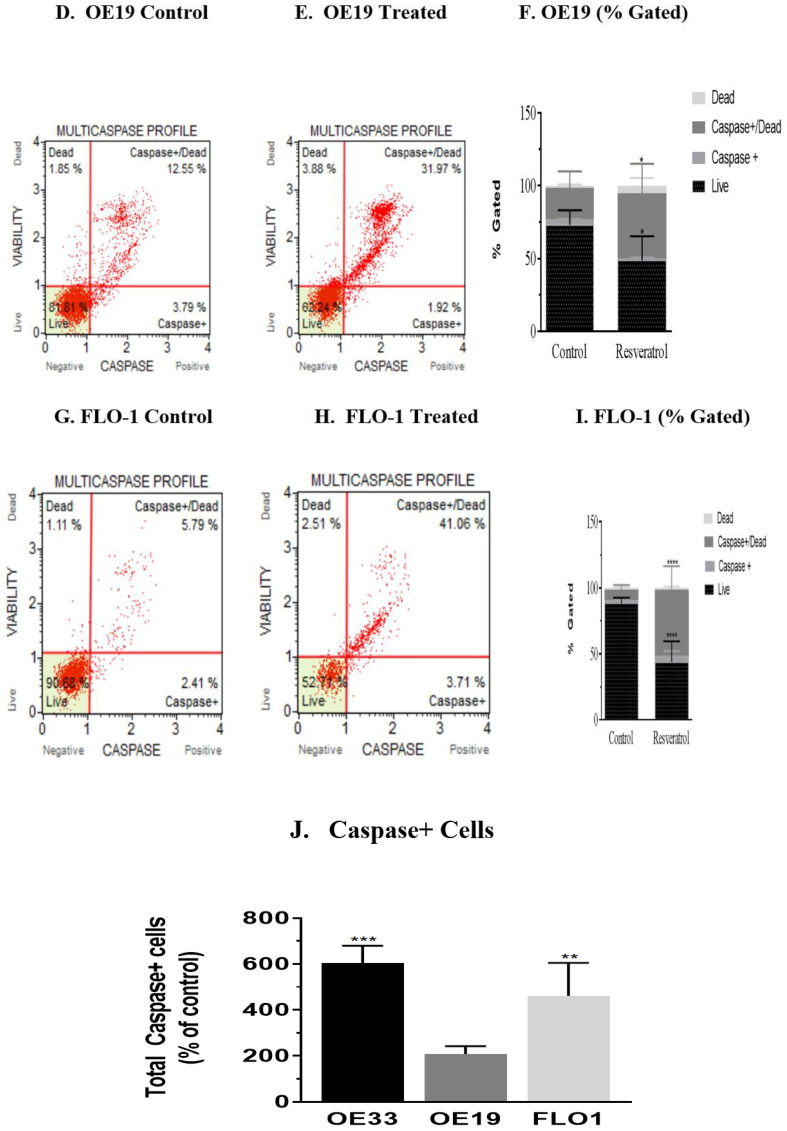
Activation of caspases on treatment with resveratrol in OE33, OE19 and FLO-1 cells. Cells were seeded at a density of 62,500 cells/well in 12-well plates and treated with resveratrol at 100 µM for OE33 cells for 72 h. Three independent experiments were performed. (**A**) Representative scatter plot from Muse MultiCaspase assay for the control group. (**B**) Representative scatter plot from Muse MultiCaspase assay for the resveratrol-treated group. (**C**) Profiles (% gated) of the control and treated group, respectively. Similarly, OE19 and FLO-1 cells were treated with resveratrol at an IC_50_ concentration of 50 and 40 μM, respectively, and analyzed by Muse flow cytometry and Muse MultiCaspase assay. Representative scatter plots of four independent trials of the control group and the treated group for OE19 cells are shown in (**D**) and (**E**), respectively. (**F**) The % gated profile of the control group and treated group for OE19 cells. Representative scatter plots of four independent trials of the control group and the treated group for FLO-1 cells are shown in (**G**) and (**H**), respectively. (**I**) The % gated profile of the control group and treated group for FLO-1 cells. (**J**) After normalizing to the control, the percent of caspase-positive cells are represented by the bar diagram for all the three cell lines. * in the bar diagram indicates *p*-value < 0.05. More asterisks represent higher statistical significance.

We sought to examine if caspases, the key players in the apoptotic pathway, were involved in causing cell death. As it has been reported that a number of caspases play a role in causing apoptosis, we decided to assay for the involvement of multiple caspases. To this end, OE33, OE19 and FLO-1 cells were treated with resveratrol at the IC_50_ concentration observed for each of the cell lines. Caspase activation was monitored by Muse flow cytometry and Muse MultiCaspase assay. Three independent trials were performed and representative scatter plots of the control group and the resveratrol-treated group (100 μM) for OE 33 cells are shown in Figure 3A,B, respectively. The profiles of the treated group versus the control group showed an increase in caspase-positive cells, see Figure 3C. Similarly, OE19 and FLO-1 cells were treated with resveratrol at an IC_50_ concentration of 50 and 40 μM, respectively, and analyzed by Muse flow cytometry and Muse MultiCaspase assay. Representative scatter plots of four independent trials of the control group and the treated group are shown in Figure 3D,E respectively, for OE19 cells. The profiles of the treated group versus the control group showed an increase in caspase-positive cells, Figure 3F. Representative scatter plot of four independent trials of the flow cytometric and MultiCaspase assay for FLO-1 control and treated cells are shown in Figure 3G,H. The profiles of the control group versus the treated group showed an increase in caspase-positive cells, as seen in Figure 3I. When normalized to the control group for each cell line, a significant increase in caspase-positive cells were observed, Figure 3J; there was an increase of >5-fold in OE33 cells, about 2-fold in OE19 and >2-fold in FLO-1 cells.

### 3.4. Resveratrol Impacts the Levels of Reactive Oxygen Species (ROS) in OE33, OE19 and FLO-1 Cells

**Figure 4:**
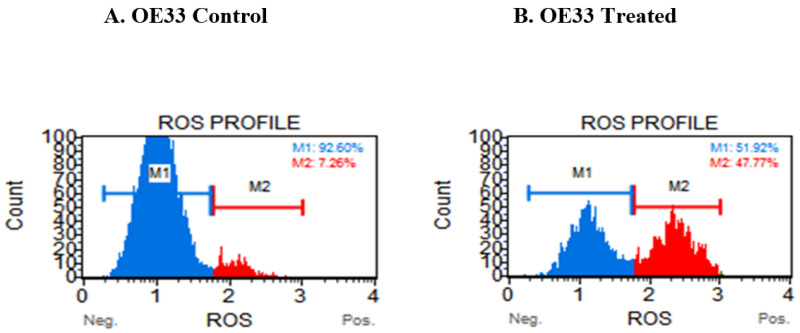

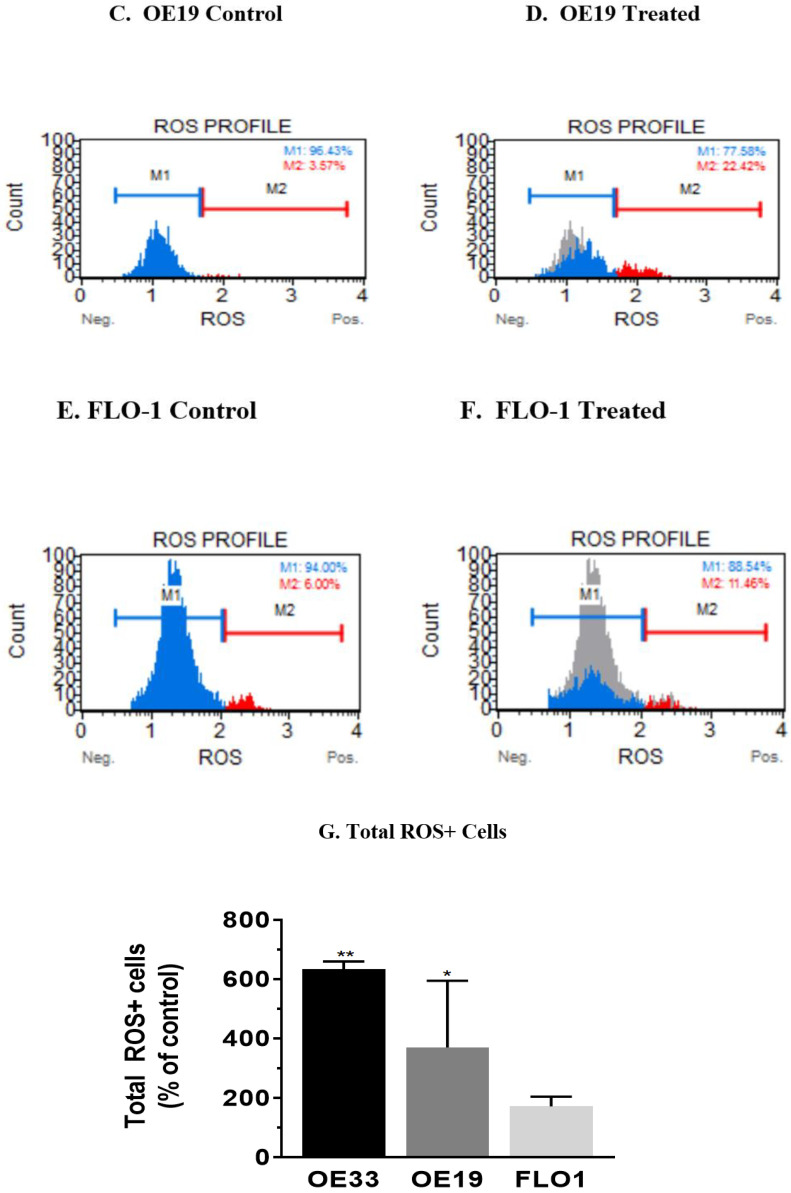
Impact of resveratrol on the levels of reactive oxygen species (ROS) in OE33, OE19 and FLO-1 cells. Cells were seeded at a density of 62,500 cells/well in 12-well plates, treated with resveratrol at 100 µM for OE33 cells and incubated for 72 h. Three independent experiments were performed. (**A**) Representative Muse Oxidative stress assay plot for the control group. (**B**) Representative plot from Muse Oxidative stress assay for resveratrol-treated OE33 group. Similar experiments were performed to assess the generation of ROS in OE19 cells on treatment with 50 μM (IC_50_ concentration) of resveratrol. (**C**) Representative Muse Oxidative stress assay plot for the control OE19 group. (**D**) Representative plot from Muse Oxidative stress assay for resveratrol-treated OE19 group. For FLO-1 cells, studies performed to assess the generation of ROS were similar to OE33 and OE19 except the concentration of resveratrol was 40 μM (IC_50_ concentration). (**E**) Representative Muse Oxidative stress assay plot for the control FLO-1 group. (**F**) Representative plot from the Muse Oxidative stress assay for resveratrol-treated FLO-1 group. The total ROS-positive cells after normalizing to the control group for each cell line are represented by the bar graph in (**G**). Four independent experiments were performed, and each time, cells were plated and treated in triplicates. * in the bar diagram represents *p*-values < 0.05 and more asterisks represent *p*-values of higher significance. M1= Ros (−) and M2 = Ros (+).

To ascertain if reactive oxygen species (ROS) was contributing to apoptosis occurring on resveratrol treatment, we monitored for the increase in the generation of ROS by utilizing the Muse oxidative stress assay. Cells were treated with IC_50_ concentration of resveratrol and production of reactive oxygen species was assessed by using the Oxidative Stress kit and Muse Cell Analyzer. The representative profile of three independent trials of ROS assessment in OE33 control cells is shown Figure 4A and ROS generated in the treated sample is shown in Figure 4B. Similarly, representative profile from four independent trials of ROS levels in the control cells of OE19 is shown in Figure 4C and treated samples in Figure 4D. Furthermore, a representative profile from four independent trials of ROS levels in the control cells of FLO-1 is shown in Figure 4E and the treated profile is shown in Figure 4F. When normalized to the control group of each cell line, an increase in ROS levels was observed, see Figure 4G. A marked increase in ROS production was observed in OE33 cells; additionally, an increase was observed in FLO-1 cells, while this was not statistically significant in the case of OE19 cells.

### 3.5. Resveratrol Affects the Bcl2 Levels in Esophageal Adenocarcinoma Cells

**Figure 5:**
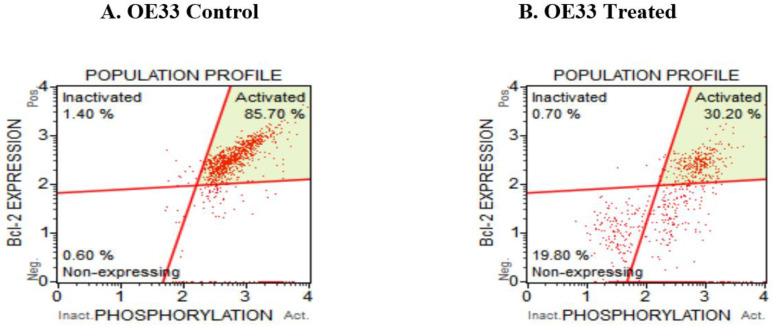

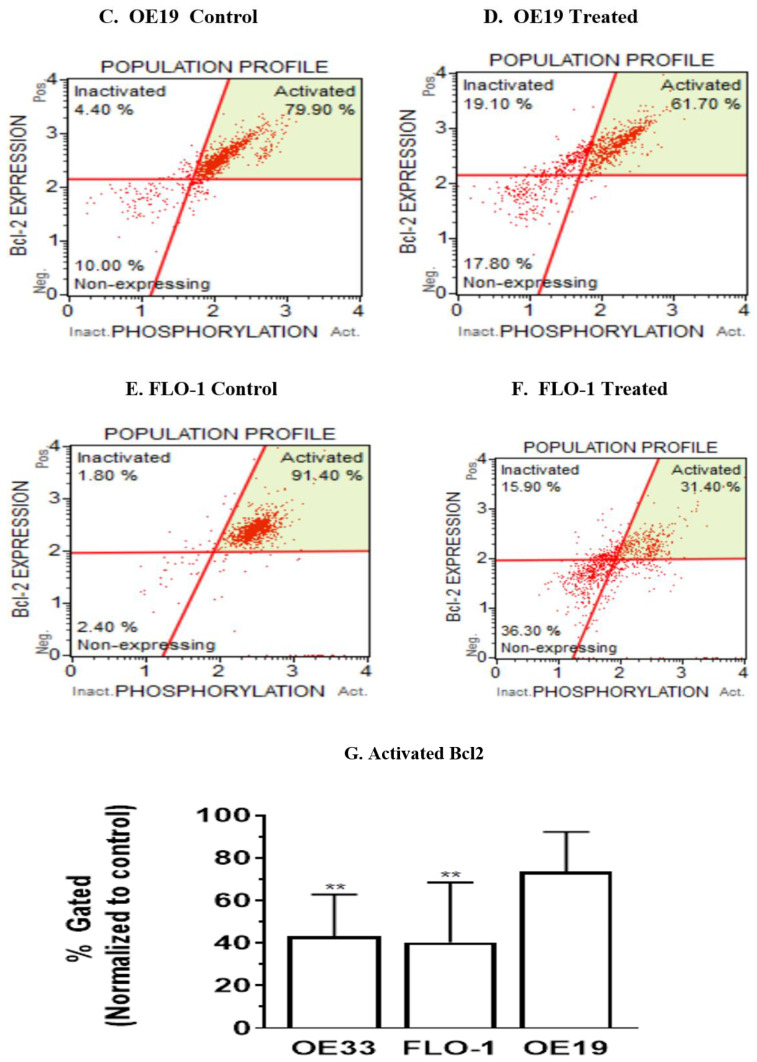

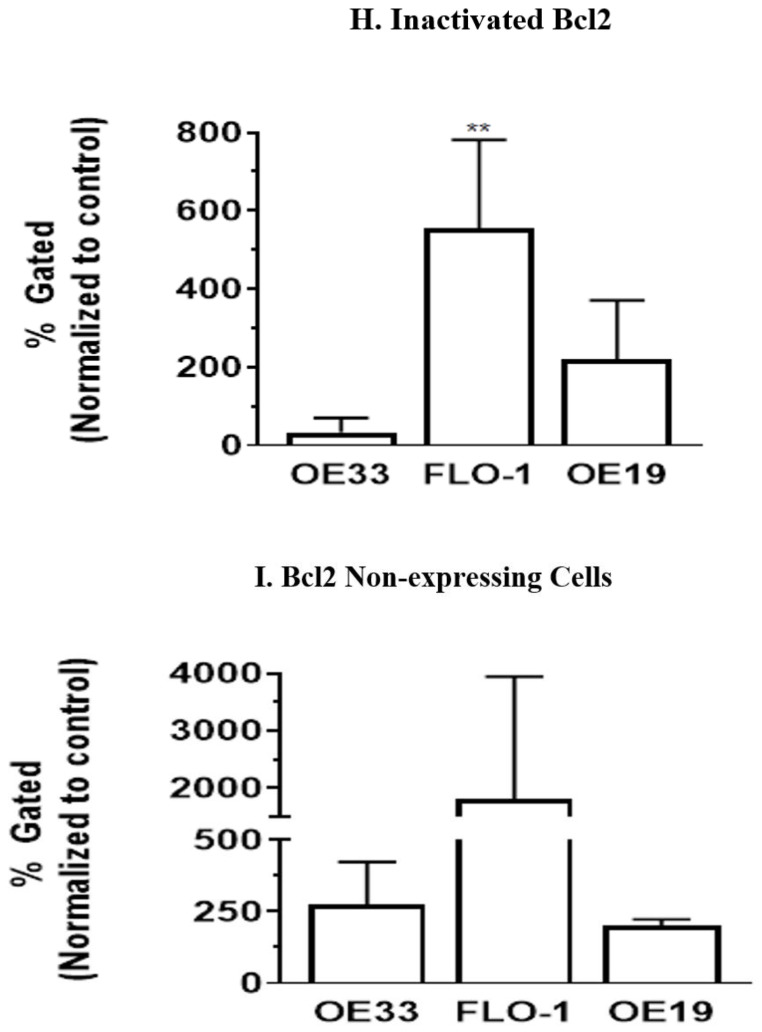
Impact on Bcl2 levels in resveratrol-treated esophageal adenocarcinoma (OE33, OE19 and FLO-1) cells. OE33 cells were grown in 12-well plates; 62,500 cells/well were seeded and they were treated with resveratrol at 100 µM for 72 h. Three independent experiments were performed. (**A**) Representative scatter plot from Muse Bcl2 dual activation assay of the control group. (**B**) Representative scatter plot from Muse Bcl2 dual activation assay of the treated OE33 group. For OE19 cells, the experiments were similar to OE33, but the resveratrol concentration was 50 µM (IC_50_ concentration). (**C**) Representative scatter plot from Muse Bcl2 dual activation assay of the control OE19 group. (**D**) Representative scatter plot from Muse Bcl2 dual activation assay of the treated OE19 group. FLO-1 cells were treated similar to the OE33 and OE19 cells, except the resveratrol concentration used was 40 µM (IC_50_ concentration). (**E**) Representative scatter plot from Muse Bcl2 dual activation assay of the control FLO-1 group. (**F**) Representative scatter plot from Muse Bcl2 dual activation assay of the treated FLO-1 group. On normalizing to the control group of each cell line, the Bcl2 levels in each cell line are shown by the bar diagram. (**G**) Bar graphs represent activated Bcl2 levels. (**H**) Inactivated molecules of Bcl2, (**I**) Bcl2 non-expressing cells. A one-way ANOVA was used using Dunnett’s multiple comparisons test with the Alpha value set to 0.05. ** indicates adjusted *p*-values between 0.01 and 0.001.

Bcl2 is a major player in the cell death/cell survival pathway, and is known to be a key molecule in various cancers; thus, we investigated if Bcl2 was affected when treating OE33, OE19 and FLO-1 cells with resveratrol. Bcl2 levels were monitored by a Muse cell analyzer using the Bcl2 assay kit. Representative scatter plot of the OE33 control sample is shown in Figure 5A and that of the treated group in Figure 5B. Similarly, OE19 cells were also treated with resveratrol and the levels of Bcl2 molecules were analyzed. Representative data (of four independent trials) of the OE19 control and treated group are shown in Figure 5C,D. Furthermore, Bcl2 levels were investigated in FLO-1 cells on treatment with resveratrol. A representative scatter plot of four trials for the control is shown in Figure 5E and similarly for the treated FLO-1 cells in Figure 5F. The data acquired showed that resveratrol treatment in OE33 cells, decreased the number of activated molecules and marginally increased the percentage of Bcl2 non-expressing cells. A moderate increase in the non-expressing cells and a decrease in the activated molecules were observed in OE19 cells. An increase in the inactivated Bcl2 molecules with a decrease in the activated Bcl2 levels were observed in resveratrol-treated FLO-1 cells, as shown in Figure 5G–I.

### 3.6. Proteomic Analysis Reveals Entities of Pivotal Signaling Pathways That Were Differentially Regulated on the Administration of Resveratrol in the Esophageal Adenocarcinoma Cell Lines (OE33, OE19 and FLO-1)

**Figure 6:**
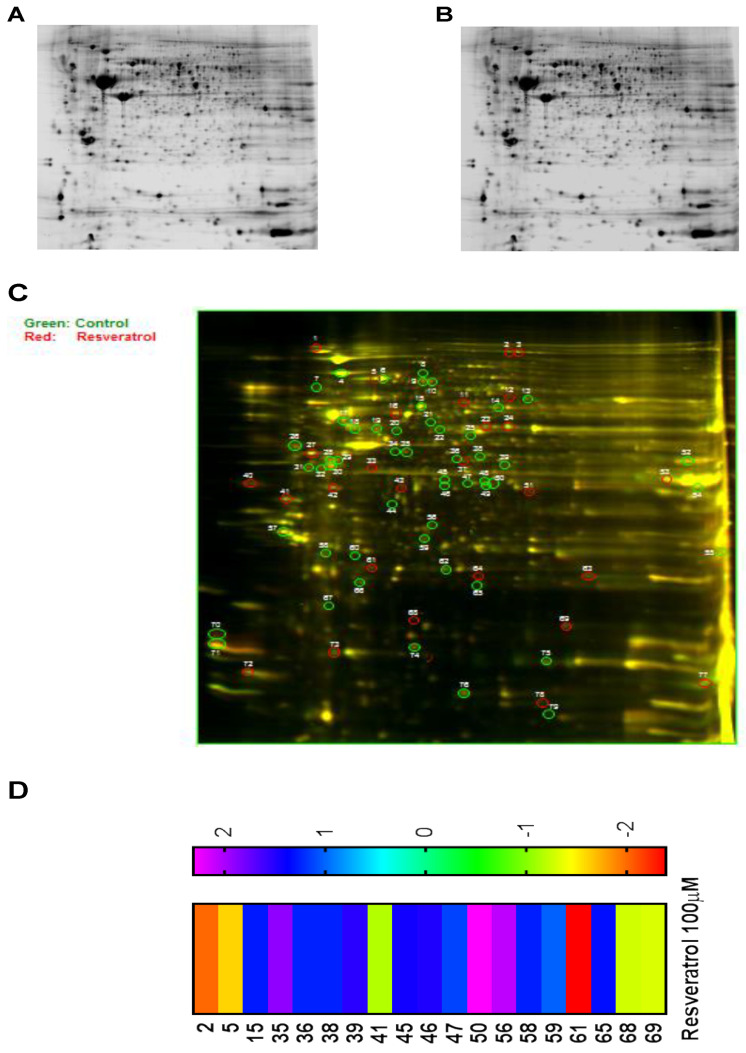
Proteomic analysis of esophageal adenocarcinoma cells (OE33, OE19 and FLO-1 cells) on treatment with resveratrol. OE33 cells were treated with the IC_50_ concentration (100 μM) of resveratrol. Representative 2D gel image of the control (**A**) and the treated sample (**B**) is shown. Cell lysates of the control group were combined with Cy3 dye and the treated group with Cy5 dye. Overlay of the two gels on isoelectric focusing (IEF) is shown (**C**). Differentially expressed proteins on the gel are circled. (**D**) Heatmap showing the fold change in the expression of the identified proteins in treated sample on comparing to the control sample. Similar proteomic analysis was performed with OE19 and FLO-1 cells on treatment with resveratrol at the IC_50_ concentration of 50 μM and 40 μM, respectively. The raw datasets from cell line OE19 and FLO-1 are provided in the Supplemental Material Figure S1. Spots common to all the three cell lines were picked and analyzed.

To gain knowledge of more entities involved in the multifaceted action of resveratrol, we opted to perform a 2-DIGE and mass spectrometric analysis, as this method of analysis could shed light on more molecules involved in the action of resveratrol. The rationale for employing 2-DIGE and mass spectrometric analysis was that this method of protein profiling is highly sensitive as specific peptides of proteins are assessed by mass spectrometry and the accuracy of detection is in the femtomol range. Two-dimensional gel electrophoresis and mass spectrometric analysis were performed with samples obtained from the treated and the control groups of OE33, OE19 and FLO-1 cells (As described in the Section 2. Representative gel pictures of both the control and the treated group of OE33 cells show clear separation of proteins, as shown in Figure 6A,B. For proteomic analysis, the control proteins were tagged with Cy3 (green fluorescence) and the treated group with Cy5 (red fluorescence) prior to being subjected to electrophoresis. An overlay of the gels exhibited the differentially regulated proteins. These proteins are shown by circles on the protein gel, as shown in Figure 6C. The number of differentially expressed proteins are shown in the heat map of proteins in Figure 6D. Based on the fold changes obtained, we selected upregulated and downregulated proteins for further proteomic analysis by mass spectrometry. OE33, OE19 and FLO-1 cells were treated with resveratrol at an IC_50_ concentration of 100 μM, 50 μM and 40 μM, respectively, and were processed in an identical manner. Moreover, based on the fold changes obtained, the upregulated and downregulated proteins were selected for further proteomic analysis by mass spectrometry.

The details of the molecules profiled with Fold Changes are represented in Table 1.

Among the downregulated proteins, elongation factor 2 was one of the entities in resveratrol-treated esophageal cells. This factor plays a pivotal role in the control of cell proliferation. Its downregulation could be contributing to the loss of viability of the three esophageal adenocarcinoma cells that we observed in the cellular analysis in the present study.

Peptidyl-prolyl cis-trans isomerase B (PPIases) are conserved family of proteins and are multifunctional. Downregulation of these essential molecules by resveratrol could lead to loss of cell viability and have an effect on molecular entities of the esophageal cells, as observed in the molecular and cellular analysis that we performed.

X-ray repair cross-complementing protein 5 (XRCC5), also termed Ku80 is an essential protein involved in repairing DNA damage along the NHEJ pathway. Downregulation of this central entity in the present study in the three cell systems studied, could have a major impact on cell proliferation, apoptosis and inhibition of key regulatory pathways operating in esophageal cancers, which are known to be aggressive in nature.

Keratin type II cytoskeletal 8 plays a major role as protector of epithelial cell integrity. Downregulation of this molecule by resveratrol in esophageal cell system in the present study could have significant effect on the cell viability that we observed in cellular level assessment.

Heterogeneous nuclear ribonucleoproteins A2/B1 (hnRNP A2/B1) are a family of proteins which are splice variants that are closely related, and therefore, they are treated together as hnRNP A2/B1. A decrease in this key entity in all the three esophageal adenocarcinoma cell systems that we observed in the present study could have a profound impact on key pathways in this type of aggressive cancer.

BH3-interacting domain death agonist (BID) has been reported to be elevated in certain tumors and has also shown to be involved in alternate apoptotic pathways. Therefore, a decrease in BID by resveratrol could alleviate other alternate pathways that could potentially operate in evading apoptosis.

Transcription factors, such as BTF3, play a central role in controlling the gene expression of cancer genes. Downregulation of this factor in the three esophageal cell lines that we observed by protein profiling could have a deleterious effect on esophageal cancer cells.

Among the upregulated proteins by resveratrol in the present study, heat shock protein HSP 90-beta was one of the proteins identified. By binding to other key molecules, it is reported to cause apoptosis. Therefore, it could be playing a role in the induction of apoptosis that we observed in the assessment of molecular mechanism of action in the present study.

Serpin H1, also termed HSP47, is known to be elevated in cancers and was one of the upregulated components in the present study. Interestingly, the Serpin family of proteins are assessed in improving DNA vaccines against cancers. An increase in this component could aid in increasing the efficacy of DNA vaccines against esophageal cancers and to pursue chemo-immunotherapy treatment.

Heterogeneous nuclear ribonucleoprotein A1 (HnRNP A1) was also upregulated. This molecule belongs to the family of RNA binding proteins, also termed RBPs. An increase in this entity is reported in cancers.

Anterior gradient protein 2 homolog was one among the upregulated moiety. This protein belongs to the protein disulfide isomerase family. One such protein disulfide isomerase termed PDIA3 has been reported in esophageal cancers and plays a multifaceted role in cancers.

Cofilin was also one among the upregulated molecule identified by proteomic profiling. There are reports of it as a therapeutic target in the treatment of cancers.

The overall data from cellular analysis, molecular mode of action assessment and proteomic profiling has shed light upon molecular and immunomodulatory signatures, which could be harnessed for developing efficacious chemo-immunotherapy as well as chemo-radiation treatments for adenocarcinoma of the esophagus—an aggressive form of cancer.

**Figure 7:**
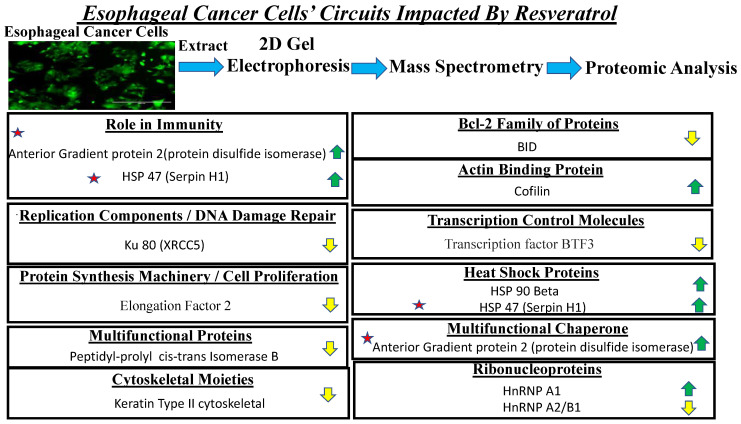
Schematic representation of pivotal circuits deregulated in the three esophageal adenocarcinoma cell lines (OE33, OE19 and FLO-1). Upregulated molecules are indicated by green arrow. Downregulated molecules are indcated by yellow arrow. Star represents the additional role of the identified entities in immunity.

In Figure 7 below, profiles of the cellular proteins and the pivotal signaling pathways that were impacted on administration of resveratrol in the three esophageal adenocarcinoma cells (OE33, OE19 and FLO-1) are represented.

## 4. Discussion

We have attempted to decipher the action of resveratrol on adenocarcinoma of the esophagus with the aim of identifying key novel targets by multi-level profiling to combat the malady. Our study was conducted in the esophageal cancer cell lines OE33, OE19 and FLO-1. In the absence of well-defined animal model system to study adenocarcinoma of the esophagus [17], we conducted in depth studies in the three model cell systems—OE33, OE19 and FLO-1. Resveratrol is a polyphenol present in natural occurring foods such as berries, grapes and wine. Here, we demonstrate by cellular analysis, the inhibition of cell viability of all the three esophageal adenocarcinoma cell systems studied on administering resveratrol.

Studies on the mechanism of action of resveratrol were performed by flow cytometry. Flow cytometric analysis has been reported to be highly sensitive [22]; further quantitative data can be obtained with this methodology. Studies performed revealed resveratrol’s impact on the programmed cell death pathway with an increase in apoptotic and caspase-positive cells. An increase in apoptotic cells on resveratrol treatment was reported in cervical cancer as well [6]. Furthermore, an increase in reactive oxygen species (ROS) was observed in OE33 and FLO-1 cells, suggesting that it also contributes to the apoptosis that we observed. Similarly, resveratrol impacts ROS and causes cell death in ovarian cancer [14]. Moreover, Bcl2 the essential entity for cell survival was impacted in the three esophageal adenocarcinoma cell lines studied. Furthermore, we have employed highly sensitive methods, such as 2DIGE and mass spectrometry, of which the accuracy is even in the fmol range [23]; additionally, these methodologies can identify modified proteins. In the current study, we focused mainly on the upregulation/downregulation of proteins to correlate with the induction of cell death as well as to explore new targets/signature molecules with therapeutic potential that are present in the three cell lines.

We sought to elucidate in detail the mode of action of resveratrol by protein profiling. Two-dimensional gel electrophoresis and mass spectrometry revealed differentially regulated proteins. We selected some of them based on the fold changes in the levels of the proteins observed in the three esophageal adenocarcinoma cell systems studied. We have categorized them based on the cellular pathways they regulate.

In the category of protein synthesis machinery, elongation factor 2 was downregulated in resveratrol-treated esophageal cells. This factor, known to control cell proliferation, was reported to be overexpressed in ovarian cancers [24], while in esophageal cancers, it is known to contribute to tumor progression and radioresistance [25]. Its activity varies based on the cell type. Downregulation of elongation factor 2 by resveratrol observed on protein profiling in the three esophageal adenocarcinoma cell system in the present study could have a potent impact on these cancers; additionally, as seen in the present study at the cellular level, significant impact on the viability of all the three esophageal cell system was observed.

Peptidyl-prolyl cis-trans isomerase B (PPIases) are conserved family of proteins and are multifunctional. They control various key activities in the cell, such as protein folding, trafficking and as chaperones. They also belong to the category of ‘Immunophilins’, and are receptors for immunosuppressant molecules. Having chemotactic and inflammatory properties, they play a role as vehicles for eosinophils, basophils and monocytes [26]. Furthermore, proteins that have PPIase activity, such as the Peptidyl-prolyl cis-trans isomerase NIMA-interacting 1 (PIN1), are shown to be upregulated in various cancers and are considered as prognostic markers and as a therapeutic target [27].

Similarly, PIN1 is overexpressed in esophageal cancers and forms a prognostic marker and a novel therapeutic target for esophageal cancer [28]. As this protein is multifunctional, the downregulation of PPIase observed in our proteomic analysis could have therapeutic implication and could be one of the factors leading to loss of cell viability and effect on molecular entities of the esophageal cells that we observed in the cellular and molecular analysis.

X-ray repair cross-complementing protein 5 (XRCC5), also termed Ku80, is an essential protein involved in repairing DNA damage along the NHEJ pathway. It is involved in multiple pivotal pathways, such as cell proliferation, telomere maintenance, apoptosis and phosphorylation of various key cellular factors including HSP90 [29]. In hepatocellular carcinoma, overexpression of Ku80 is shown to lead to metastasis [30]. Notably, in patients with superficial esophageal squamous cell carcinoma, overexpression of Ku80 led to poor prognosis [31]. Furthermore, in Ku-deficient cells, PCNA dissociates from the chromosome and Ku is required to maintain PCNA on the chromosome [32].

Moreover, in Head and Neck cancer cell lines, the sensitivity to radiation was shown to be correlating with levels of Ku80 [33]. It also plays a central role in key pathways such as DNA repair via non-homologous end joining (NHEJ), including effects on telomere maintenance [34]. We envision that treatment with resveratrol during radiation therapy of esophageal cancers could possibly prevent radioresistance and lead to efficacious treatment for esophageal cancers, which are known to be aggressive in nature.

Keratin type II cytoskeletal 8 plays a major role as protector of epithelial cell integrity. Most adenocarcinomas express cytoskeletal 8. If keratin expression is aberrant, it is known to lead to drug resistance. Keratins play a pivotal role in motility, growth and protein synthesis as well. As keratins play a major role in cancer, they could be considered not only as diagnostic and prognostic markers but as therapeutic targets as well [35]. In esophageal cancers, it is reported that the expression of cytoskeletal 8 was altered in high-risk populations and could form a useful marker for high-risk versus low-risk populations and also forms a biomarker for the early detection of esophageal cancer [36]. Downregulation of this molecule by resveratrol in the esophageal cell system in the present study could have a deleterious effect on these cells, leading to the loss of cell viability that we observed in cellular level assessment.

Heterogeneous nuclear ribonucleoproteins A2/B1 (hnRNP A2/B1) are a family of proteins which are splice variants that are closely related and differ only by 12 amino acids, and thus, they are treated together as hnRNP A2/B1. These are a group of proteins that bind RNA and are also termed RBP. They play a central role in controlling various signaling pathways, including transcription, DNA repair, mRNA metabolism, splicing, transport-RNA shuttling and modifications at the post transcriptional level, as well as being involved in the biogenesis of telomeres at the ends of DNA. Therefore, deregulation of these proteins would play a pivotal role in cell pathological conditions. In lung cancer such as NCSLC, upregulation of A2/B1 is reported [37]. In fact, elevated levels of the B1 protein are reported in esophageal carcinoma [38]. Furthermore, knockdown of hnRNP A2/B1 in glioblastoma cells led to a decrease in growth, apoptosis and ROS production [39]. Our proteomic profiling revealed a decrease in this key entity in all the three esophageal adenocarcinoma cell system studied, and could have impacted the cell viability and also could have caused the increase in apoptotic cells and ROS that we observed in the present study when performing the molecular level analysis. Therefore, this could have profound implications for resveratrol’s effect on key pathways in this aggressive cancer.

BH3-interacting domain death agonist (BID) has been reported by immunohistochemical studies to be elevated in certain tumors such as cancers of the prostrate, colon, brain tumors such as gliomas and in lymphomas. Interestingly, higher amounts were detected in advanced tumors of prostrate and lymphomas. It is envisaged that alternate apoptosis mechanisms might be contributed to high levels of BID [40]. In our studies with the esophageal cancer cell system, we found a decrease in BID in the three cell systems. Therefore, decrease in BID by resveratrol could alleviate other alternate pathways that could potentially operate in evading apoptosis. In fact, we observed an increase in apoptotic cells in the three-adenocarcinoma cell system in the present mechanism of action studies.

Control of gene expression by transcription factors plays a key role in neoplasia. Transcription factors, such as BTF3, are shown to control tumor-associated genes in pancreatic cancers [41]. In colon cancer, downregulation of BTF3 resulted in the inhibition of cell proliferation with cells in the early apoptotic phase [42]. Moreover, resveratrol has been reported to modulate transcription factors [12]. A decrease in BTF3 by resveratrol, which controls cancer-associated gene transcription, could be pursued for inhibiting cell proliferation of adenocarcinoma of the esophagus, leading to the apoptosis of these cells, as seen in the molecular analysis in the present study.

Among the upregulated proteins by resveratrol in the present study, heat shock protein HSP 90-beta was one of the proteins identified. HSP 90 is a cell chaperone playing an important role in cell survival and is a chemotherapeutic target for cancers. There are reports of its role as a proapoptotic molecule as well; it was found that in leukemic cells, HSP90 interacts with c-Jun N- terminal kinase (JNK) in lipid rafts when Edelfosine was administered as an antileukemic therapy to induce the apoptotic response [43]. In fact, in colon cancer cells, resveratrol induces apoptosis through the redistribution of Fas in the rafts, leading to death-inducing signaling complex [44]. Thus, resveratrol-induced apoptosis is associated with Fas redistribution in the rafts and the formation of a death-inducing signaling complex in colon cancer cells. Additionally, it is reported that Fas and JNK are located on lipid rafts [45]. In our present study, as we have administered resveratrol, we envision that there could be a possibility of the upregulated HSP 90 that we identified in the proteomic analysis to be exhibiting a proapoptotic action, in light of the report that one of the actions of resveratrol is through lipid rafts, as seen in colon cancer cells. Thus, it could be playing a role in the induction of apoptosis that we observed in the assessment of molecular mechanism of action in our present study.

Serpin H1, also termed HSP47, is known to be elevated in cancers. We observed upregulation of this entity on resveratrol treatment in the three esophageal adenocarcinoma cell lines studied. Interestingly, the mouse homolog Spi6 of human serpin P19 has been reported to have an effect on CD8-T cell responses by having an effect on the DC antigen-presenting cell’s viability [46]. Notably, to improve the effect of DNA tumor vaccines, there is a need to increase the viability of dendritic cells, and a preliminary study in mice has shown that this could be achieved by using mouse serpin DNA as an adjuvant [47]. Therefore, an increase in this entity in resveratrol-treated esophageal cells could be harnessed to improve anti-tumor DNA vaccines against esophageal cancers to increase efficacy of treatment employing the strategy of chemo-immunotherapy. Another possibility could be the use of micro RNAs that target serpins (HSP 47), as reported for cervical cancer [48]. This could be considered under combination therapy for esophageal cancers.

Heterogeneous nuclear ribonucleoprotein A1 (HnRNP A1) belongs to the family of RNA binding proteins also termed RBPs. HnRNP A1 in particular is shown to be involved in lung cancer proliferation [49] Although hnRNP A1 was upregulated in our present study, hnRNP A2/B1 was downregulated. It has been shown in cervical cancer that inhibition of hnRNP A2/B1 inhibits cell proliferation and triggers apoptosis [50]; additionally, in this cell line, it has been shown that knockdown of hnRNP A2/B1 markedly reduced the proliferation of these cells accompanied by a reduction in PCNA and Ki-67. Therefore, the downregulation of this hnRNP A2/B1 by resveratrol in the present study in the three esophageal cell systems could have a profound effect on curbing the growth potential of esophageal cancers, because pivotal entities such as PCNA and Ki 67 are shown to be downregulated by this entity.

Anterior gradient protein 2 homolog belongs to the protein disulfide isomerase family [51]. One of the proteins in the family of disulfide isomerase, termed PDIA3, is considered as a prognostic marker in esophageal cancer and also in other cancers, such as gastric cancer [52,53]; the survival prospect is favorable when the PDIA3 level was high when compared to when it was low. Additionally, protein disulfide isomerases are known to control key regulatory processes in a cell. PDIA3 is a chaperone protein which is involved in the folding and processing of proteins on their synthesis in the cytoplasm. It is known to have a proapoptotic function [54]. We envision that upregulation of the ‘Anterior gradient protein 2 homolog’ belonging to the protein disulfide isomerase family observed by protein profiling in the present study could contribute towards apoptosis, as observed in our molecular studies. Notably, PDIA3 is involved in antigen processing as well. On immunoprecipitation, the MHC complex I immunoprecipitated along with PDIA3, shedding light upon its function in the immune pathway. Additionally, PDIA3 has been described as tumor-associated antigen (TAA) in colon cancer patients; furthermore, autoantibodies in these patients resulted in a specific and efficacious T cell response [55]. Similar to colon cancer, in NCSLC patients, an increase in calreticulin (CALR) along with PDIA3 predicted better prognosis. CALR together with PDIA3 has been shown to translocate from the ER to the cell surface, leading to the recognition of the tumor by dendritic cells and then to T cell mediated eradication of the tumor [56]. We envision that upregulation of anterior gradient protein 2 homolog belonging to the protein disulfide isomerase family by resveratrol in the esophageal adenocarcinoma cells in the present study could be beneficial and aid an immune attack on esophageal cancers.

Cofilin was also one among the entities upregulated by resveratrol. Overexpression of Cofilin is considered to lead to the invasion and metastasis in certain cancer cell lines [57]; however, in other cancer cell systems, its overexpression is considered to counteract invasion [58]. However, recent evidence suggests that it is not a single gene such as cofilin but the entities of the entire cofilin pathway which could be governing the invasive property of tumor cells [59]. Involvement of cofilin in apoptosis has become a major focus for the treatment of cancers recently, because active (dephosphorylated) cofilin is known to affect the mitochondrial function and lead to the release of cytochrome C [60]. Therefore, cofilin could not only serve as a biomarker, but also as a viable novel therapeutic target for esophageal adenocarcinomas, which are aggressive forms of cancer.

In our present study, the observed differences in sensitivity to resveratrol could be due to the varied cancer driver gene status in the cell lines. Apart from cancer driver genes such as TP53 and Myc, the expression of the PSMD3 gene, which codes for the 19S component of the proteasomes, differs in the three cell lines [61]. Additionally, amplification of genes such as Aurora Kinase has been shown to confer resistance to chemotherapeutics [62]. It has been reported that OE33 has a 5-fold amplified Aurora kinase gene, whereas FLO-1 and OE19 have an amplification of 3.5- and 1.7-fold [63], respectively, which could also be contributing to the differences in responses observed in our study.

## 5. Conclusions

In conclusion, the phenotypic outcome observed with resveratrol could be based on the stoichiometry of the fold changes per se of some of the pivotal entities identified in our study. Based on the proteomic analysis, DNA repair, DNA replication, transcription, cytoskeletal moieties, actin binding component and proliferation pathways were impacted. In the future, compounds that are synergistic with resveratrol could be pursued as well in a combinatorial approach. Importantly, proteomic profiling on resveratrol treatment has shed light on the pivotal cell signaling pathways and has highlighted molecular and immunomodulatory signatures such as Serpin (HSP47) and Anterior gradient protein 2- a protein disulfide isomerase with implications for chemo-immunotherapy as well as the development of anticancer vaccines for adenocarcinoma of the esophagus. Moreover, the downregulation of the pivotal entity Ku80 in the three-cell system studied could have a major impact in decreasing resistance to radiation therapy in adenocarcinomas of the esophagus, which are known to be highly aggressive in nature. Therefore, further studies are warranted with resveratrol as a treatment for esophageal adenocarcinomas.

## Figures and Tables

**Table 1:** Differentially Regulated Entities on Resveratrol Treatment in OE33, OE19 and FLO-1.

SI#	Protein	Fold Change in OE33	Fold Change in OE19	Fold Change in FLO-1
1	Elongation Factor 2	−1.83	−1.97	−1.94
2	X-ray repair cross-complementing protein 5	−2.05	−2.25	−1.76
3	Keratin type II cytoskeletal 8	−6.86	−7.48	−2.22
4	Heterogenous nuclear ribonucleoproteins A2/B1	−1.9	−3.8	−1.3
5	BH3-interacting domain death agonist	−1.66	−2.03	−1.6
6	Transcription factor BTF3	−2.31	−4.82	−1.29
7	Peptidyl prolyl cis-trans isomerase B	−1.55	−1.92	−5.27
8	Heat shock protein HSP 90-beta	2.00	1.74	1.98
9	Serpin H1	3.06	1.35	1.75
10	Heterogenous nuclear ribonucleoprotein A1	3.25	2.21	2.51
11	Cofilin-1	2.13	3.25	2.33
12	Anterior gradient protein 2 homolog	2.18	2.97	2.2

## Data Availability

All the data relating to this article are presented in the manuscript.

## Supplementary Materials

The following are available online at https://www.mdpi.com/article/10.3390/cancers13225811/s1, Figure S1: Raw datasets from proteomics analysis for OE19 and FLO-1 cells.
