# Supplementary material for: Interception of Signaling Circuits of Esophageal Adenocarcinoma Cells by Resveratrol Reveals Molecular and Immunomodulatory Signatures
Source: Cancers (Basel). 2021 Nov 19;13(22):5811. doi: 10.3390/cancers13225811 (PMC8616317; doi:10.3390/cancers13225811)
Supplement: Supplementary file 1 [file cancers-13-05811-s001.zip › cancers-1434702-supplementary.pdf]

FLO-1

Control

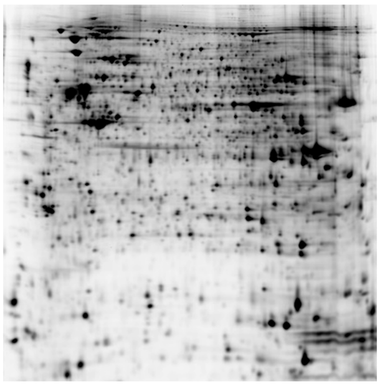

Resveratrol

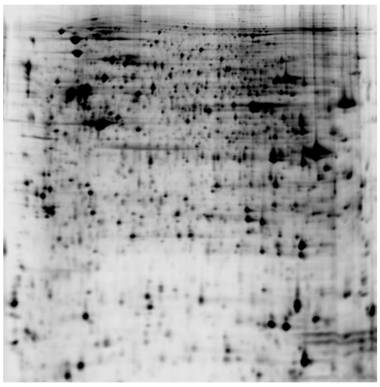

Overlay

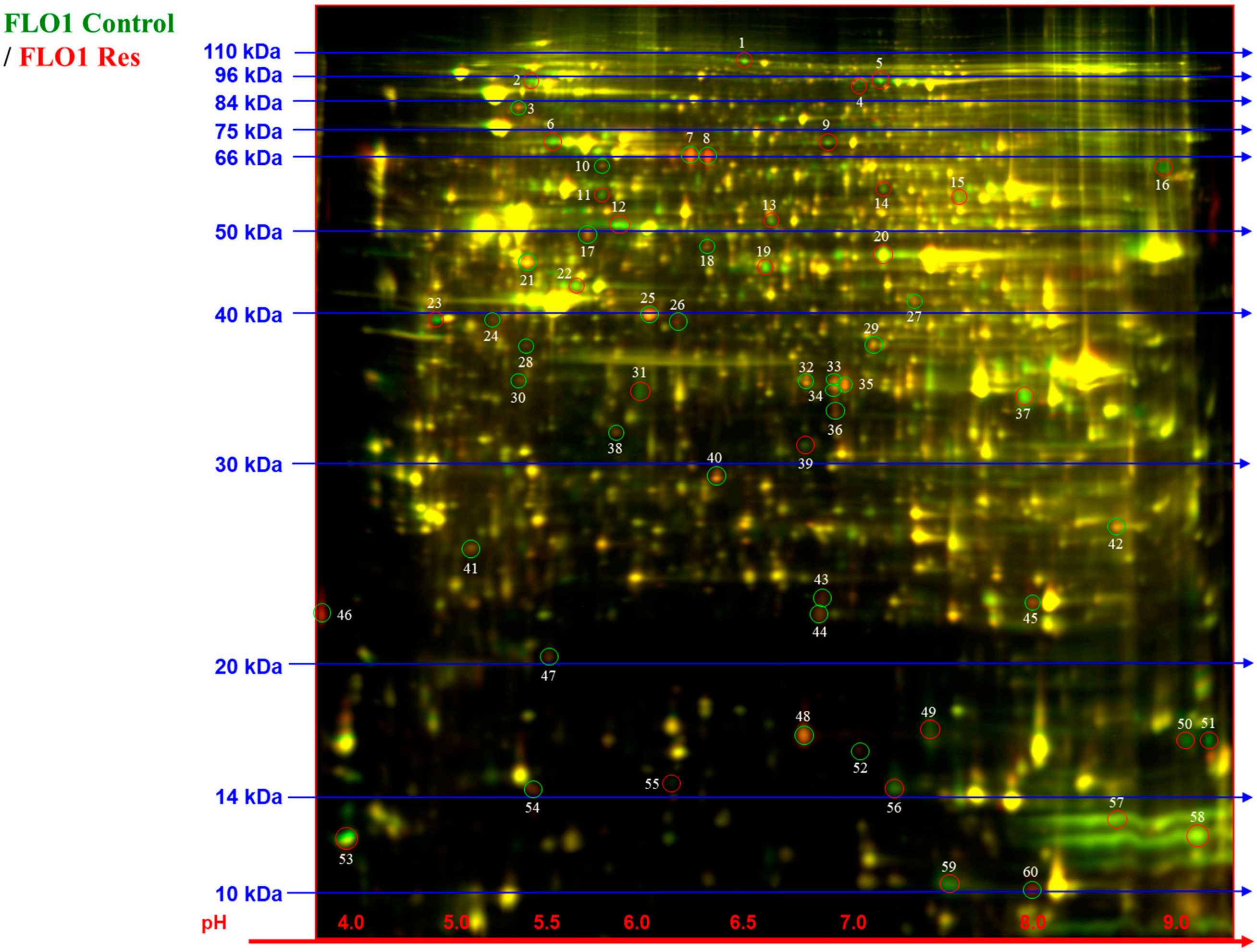

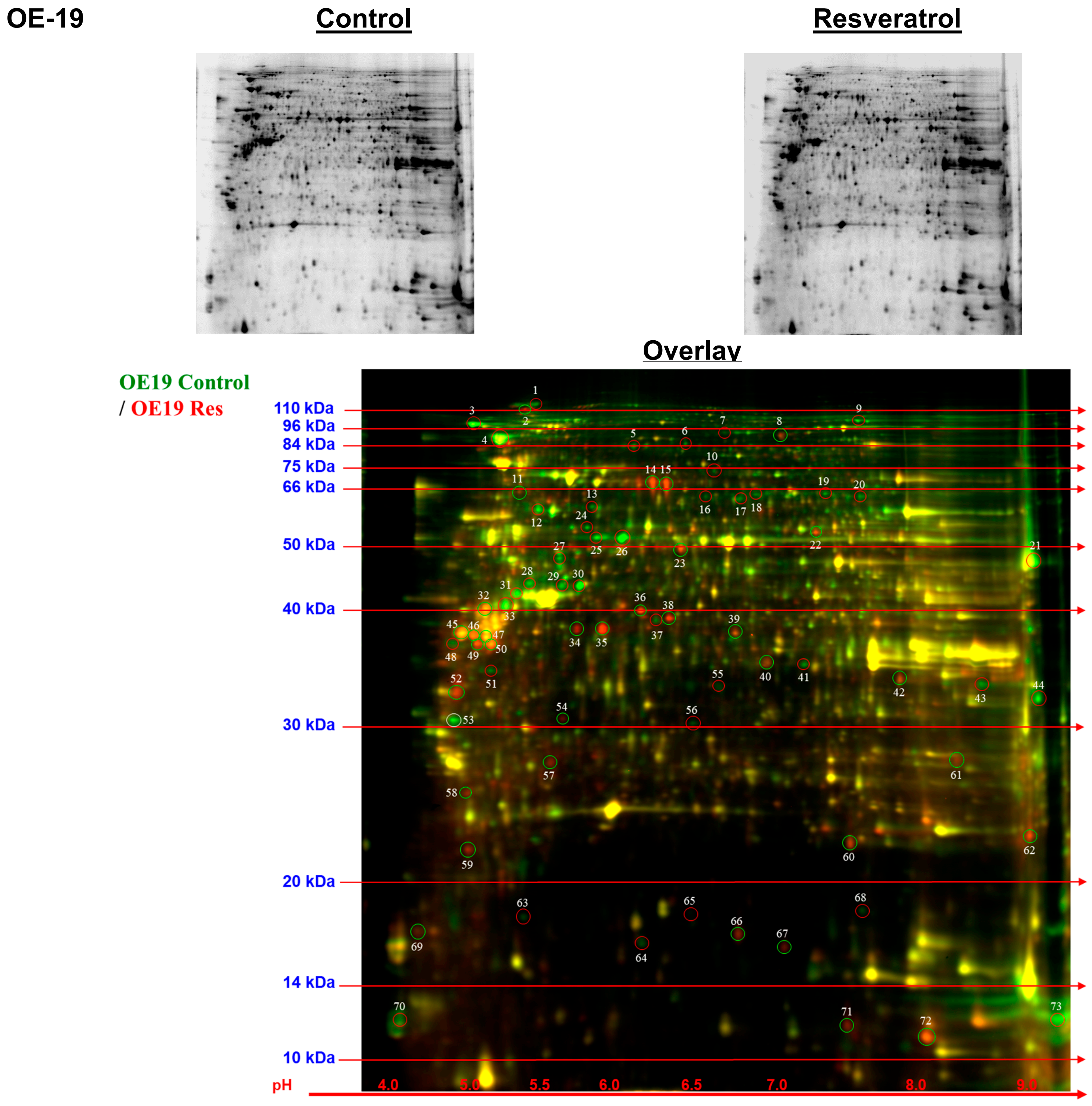

Figure S1. Proteomic Analysis of OE19 and FLO-1 cells on treatment with resveratrol at the IC_{50} concentration of 50 µM and 40 µM, respectively. Differentially expressed proteins are circled. Detailed procedures are described in Materials and Methods.
